# The Experience of Health Professionals With Misinformation and Its Impact on Their Job Practice: Qualitative Interview Study

**DOI:** 10.2196/38794

**Published:** 2022-11-02

**Authors:** Nashwa Ismail, Dhouha Kbaier, Tracie Farrell, Annemarie Kane

**Affiliations:** 1 School of Education Durham University Durham United Kingdom; 2 School of Computing and Communications The Open University Milton Keynes United Kingdom; 3 Knowledge Media Institute The Open University Milton Keynes United Kingdom; 4 Faculty of Arts and Social Sciences The Open University Milton Keynes United Kingdom

**Keywords:** health misinformation, social media, health professional, patients, trust, communication, COVID-19, intervention, qualitative research, interpretive phenomenological analysis, thematic analysis, misinformation, health practitioner, infodemiology

## Abstract

**Background:**

Misinformation is often disseminated through social media, where information is spread rapidly and easily. Misinformation affects many patients' decisions to follow a treatment prescribed by health professionals (HPs). For example, chronic patients (eg, those with diabetes) may not follow their prescribed treatment plans. During the recent pandemic, misinformed people rejected COVID-19 vaccines and public health measures, such as masking and physical distancing, and used unproven treatments.

**Objective:**

This study investigated the impact of health-threatening misinformation on the practices of health care professionals in the United Kingdom, especially during the outbreaks of diseases where a great amount of health-threatening misinformation is produced and released. The study examined the misinformation surrounding the COVID-19 outbreak to determine how it may have impacted practitioners' perceptions of misinformation and how that may have influenced their practice. In particular, this study explored the answers to the following questions: How do HPs react when they learn that a patient has been misinformed? What misinformation do they believe has the greatest impact on medical practice? What aspects of change and intervention in HPs' practice are in response to misinformation?

**Methods:**

This research followed a qualitative approach to collect rich data from a smaller subset of health care practitioners working in the United Kingdom. Data were collected through 1-to-1 online interviews with 13 health practitioners, including junior and senior physicians and nurses in the United Kingdom.

**Results:**

Research findings indicated that HPs view misinformation in different ways according to the scenario in which it occurs. Some HPs consider it to be an acute incident exacerbated by the pandemic, while others see it as an ongoing phenomenon (always present) and address it as part of their daily work. HPs are developing pathways for dealing with misinformation. Two main pathways were identified: first, to educate the patient through coaching, advising, or patronizing and, second, to devote resources, such as time and effort, to facilitate 2-way communication between the patient and the health care provider through listening and talking to them.

**Conclusions:**

HPs do not receive the confidence they deserve from patients. The lack of trust in health care practitioners has been attributed to several factors, including (1) trusting alternative sources of information (eg, social media) (2) patients' doubts about HPs' experience (eg, a junior doctor with limited experience), and (3) limited time and availability for patients, especially during the pandemic. There are 2 dimensions of trust: patient-HP trust and patient-information trust. There are 2 necessary actions to address the issue of lack of trust in these dimensions: (1) building trust and (2) maintaining trust. The main recommendations of the HPs are to listen to patients, give them more time, and seek evidence-based resources.

## Introduction

### Background

Health misinformation is currently recognized as a significant issue [1-3]. Misinformation has been defined as information that has no scientific evidence to support it and is contradictory to the most recent, most reliable evidence [4,5]. Wang et al [3] distinguish further between *misinformation* and *disinformation* posted online and on social media platforms in particular. According to a study published by the Council of Europe recently [6], the 2 terms are defined in terms of intent to harm. *Misinformation* occurs when inaccurate information is disseminated, and it was not *intended* to cause harm. Misinformation that is *intended* to harm is called *disinformation*. False propaganda containing harassment, hate speech, and an intent to harm is considered *malinformation* [6]. Obviously, the spread of misinformation is not new but dates back to the early days of printing [3], and concerns about fake news and misinformation in traditional media have been prevalent since the early decades of the 20th century [7]. With the advent of digital technology and the internet, misinformation and disinformation have significantly changed how they are communicated worldwide and amplified rapidly. For health professionals (HPs), digital technology and the internet can play a critical role in combatting medical misinformation but have not got an opportunity to fully address medical misinformation.

During the COVID-19 pandemic, misinformation has been increasingly presented, based on anecdotal evidence, false information, or misleading information due to a lack of existing scientific knowledge. This information is false but not created with the intention of causing harm [8]. However, the onslaught of misinformation can lead to risky behaviors or reduced trust in authorities. In addition to investigating ways to identify and counter misinformation, researchers have focused on the consumers of misinformation [9], not only as recipients, but also as potential amplifiers. Who is being misinformed, and what does it mean to be misinformed?

Certain personal characteristics or demographic features have been implicated in the spread of misinformation, for example, the characteristics of extroversion and cooperativeness [10,11], dogmatism and religious beliefs [12], and overconfidence in one’s knowledge and critical analytic skills [13]. With regard to demographic features, Guess et al [14] found that conservatives are more likely to share news from fake news sources, such as Facebook, and are 7 times more likely to share news from fake domains. Guess et al [14] differentiated Facebook as an example of an echo chamber where beliefs are amplified or reinforced by communication and repetition inside a closed system. Cinelli et al [15] argued that echo chambers limit exposure to diverse perspectives and favor and reinforce presupposed narratives and ideologies. There also exist fake domains (eg, articles and websites with unknown sources). The term “fake domain” refers to a situation in which an adversary creates a fake website or social media profile for a variety of reasons, including creating confusion among a targeted community. More recently, Guess et al [16] addressed the opposite, as sharing information via Facebook was a relatively rare activity. It was found that conservatives are more likely to share articles from fake domains. Consequently, such studies can be difficult to interpret. The same study, for example, determined that older users are also more likely to share facts. Studies on the role of partisan thinking and misinformation have also had mixed results. Some studies show that conservatives share more misinformation [17,18], while other studies have argued that this correlation may be related to other potentially confounding factors, such as perceived bias in the media [19] or shared information processing tendencies of conservative versus liberal individuals [20]. Harper and Baguley [20] demonstrated that liberals and conservatives are equally vulnerable to believing misinformation but for different reasons. The authors found that the greater the partisan attachment (on either side), the more willing individuals appear to be in engaging in “cognitive distortion” to protect their views. Even whole communities of individuals can be misinformed due to their exclusion from mainstream society, such as migrant networks [21] or niche online communities [22].

This study investigates disease outbreaks where a large amount of health misinformation is produced and released. We focused on the misinformation that is related to HPs’ specialist areas using COVID-19 as a case study of a disease outbreak (1) to investigate how misinformation may have impacted health practitioners’ job practice, how they witnessed it occurring, and how they interpreted it, intervened, and responded to it and (2) to examine whether any reshaping of practices occurred or was expected to occur in response to misinformed patients. Due to the uncertainty regarding the sources of misinformation in this study, we considered all sources of misinformation, including echo chambers both online (eg, Facebook) and offline (eg, committees in religious worship places, such as mosques and churches).

### Health Professionals’ Terminology in This Study

Various terms are used in the literature to describe staff members who work in the health sector and provide health services to patients. For this research, we combined definitions from Medscape [23] and the National Health Service (NHS) [24] to determine the job description of individuals in the health sector who can serve the purpose of the study. According to Medscape [23], an HP is a provider of health care treatment and advice based on formal training and experience. The field includes those who work as nurses and physicians of all specialties and those who perform services in allied health professions. Public health and community health experts are also health professionals (HPs). According to the NHS, allied health professionals (AHPs) also include 14 categories (eg, osteopaths) that provide solution-focused, goal-centered care to support patients’ independence and help them with day-to-day living. Therefore, in this study, HPs include these 2 groups under the condition of having 1-to-1 communication and discussions with the patients. More details about the inclusion and exclusion of participants can be found in the Methods section.

### Research Questions

This qualitative study specifically explored responses to the following research questions (RQs):

- RQ1: How do HPs react when they learn that a patient has been misinformed?
- RQ2: What misinformation do they believe has the greatest impact on medical practice?
- RQ3: What aspects of change and intervention in HPs’ practice are in response to misinformation?

Due to the many potential variables influencing health practitioners’ experience and recommendations around dealing with misinformation, this research was determined to be best served by a qualitative approach, allowing us to collect rich data from a smaller subset of health care practitioners working in the United Kingdom.

## Methods

### Methodology

This study used a qualitative approach that shows the data findings and results from semistructured interviews that were conducted with HPs and that were audio-recorded. The interviews lasted between 30 and 45 minutes, with an average duration of 40 minutes. HPs included doctors and nurses from different areas in the United Kingdom, as well as 1 HP from the United States. Participant interviews were conducted either online (Teams or Zoom) or via phone call at the participant’s convenience.

### Ethical Considerations

The study was approved by the Human Research Ethics Committee at the Open University (reference no. HREC/3960/Kbaier, approval date April 14, 2021). The conducted interviews followed a predefined semistructured interview protocol informed by the study’s aims and objectives.

### Data Collection

**Figure 1:**
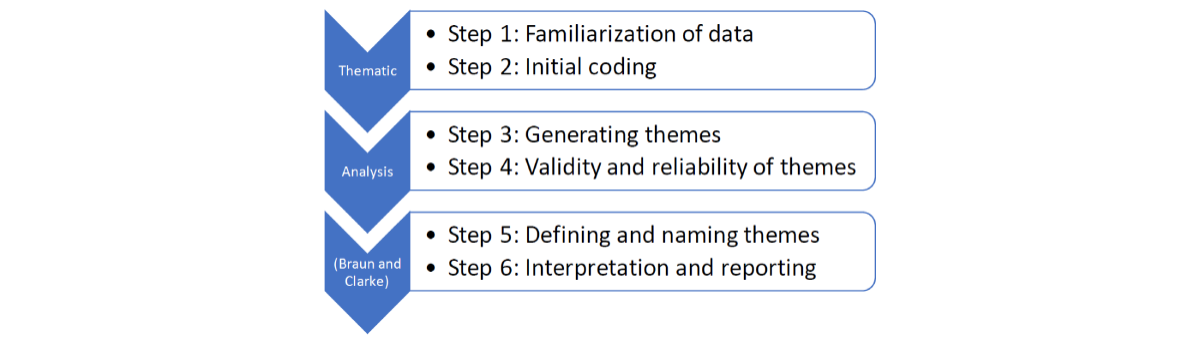
Framework for conducting a TA. TA: thematic analysis.

Data were collected through 1-to-1 online interviews (Zoom/Teams/online) with 13 health practitioners, including junior and senior physicians as well as nurses. These interviews were transcribed and analyzed using the thematic analysis (TA) approach to understand and interpret the perceived experiences of HPs during COVID-19. Participants in the study were recruited via a combination of convenience and snowball sampling methods. The HPs’ job titles were doctors and nurses. The doctors were a mix of men and women. Their professional experience ranged from junior to senior positions in different specialties, such as psychiatry and hematology. Geographically, the majority of HPs (n=12, 92%) were from the United Kingdom, except for 1 (8%) from the United States. It was useful to include a non-UK perspective to enhance understanding of the contextual factors. The HPs from the United Kingdom worked in various regional locations, including the Southeast, the Southwest, and the Midlands. Figure 1 illustrates the applied framework to analyze the qualitative data thematically, starting with reading and familiarization with the narrative across interviews, progressing to reporting the study results [25].

### Recruitment of Participants

The research team contacted (by email or telephone) the communities where the target population work in the health sector (eg, doctors and nurses in general practice in their local areas). These individuals were then asked to suggest other prospective colleagues for the study. This method is referred to as snowball sampling [26] because (in theory) once the ball starts rolling, it picks up more “snow” along the way (recruits more individuals) and the sample size grows progressively larger. In this research, the snowball sampling process consisted of 2 steps:

- Identifying potential participants: HPs they may work with or know in their workplace
- Asking those HPs to recruit other people (and then asking those people to recruit others and so on)

### Inclusion Criteria

Participants were selected based on 3 main criteria:

- Participants were currently working in the health care sector, with a job description that requires direct, day-to-day interaction, including discussions with patients. For example, staff members who were working in data entry without having any contact with patients were excluded.
- Participants have witnessed misinformation regardless of how they define what misinformation is.
- Participants’ experience extended to the period before and during the pandemic.

It is important to emphasize that misinformation that participants narrated their experience with is not about COVID-19 but about their own specialist area, as the research investigated the impact of misinformation more generally using the pandemic as a practical example of a disease outbreak. For example, an endocrine doctor narrates their experience about patients being misinformed by a family member to eat a specific fruit, believing that it would lower the level of blood sugar. In this example, the HP explained that in response to the lockdown during the pandemic, patients did not get an opportunity to have frequent face-to-face meetings with the allocated doctors and that justifies the reason for misinformation from the HP’s view.

Participants who expressed their interest in taking part in the research were then sent a participant information sheet (PIS) to explain the purpose of the research, what participants will be required to do, and how they will be involved. In addition, they were asked to sign a written consent form and send it back to the researchers. Within this consent, they were informed that the interview would be audio-recorded, and they were given the choice of whether to continue their participation in the research interviews and how to withdraw, if they wished. Finally, they were sent an email with an invitation to the interview.

### Interrater Reliability

**Figure 2:**
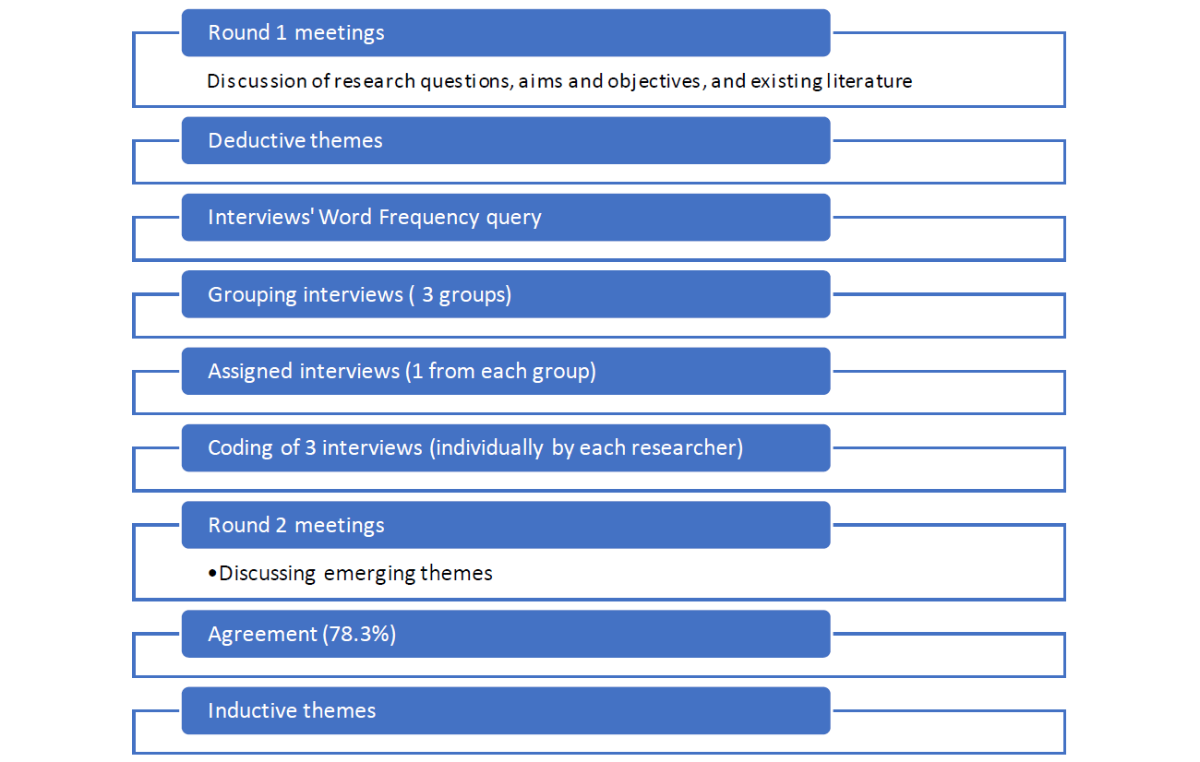
Applied steps in the IRR exercise. IRR: interrater reliability.

Figure 2 illustrates the different steps involved in the interrater reliability (IRR) process. The 2 researchers coded and analyzed the 3 agreed interviews independently, followed by comparing their coding results (superthemes and subthemes) to highlight any agreements and disagreements on the sufficiency and adequacy of the baseline data [27]. An important component of qualitative research is the identification and negotiation of disagreements throughout the iterative process of developing a codebook [28].

Reliability is calculated as the number of agreements divided by the total number of agreements and disagreements. Both node structures between the 2 coders were compared as follows:

- Score the same existing theme in both node structures as “1 agreement.”
- Score nonexisting themes in both node structures as “1 disagreement.”
- Add up all agreed themes versus adding up disagreed themes and calculate the percentage difference to determine the percentage of agreement between the 2 researchers.

We (researchers/coders) obtained a result of 78.3% agreement in our approach. Miles and Huberman [27] recommend that when coding 50 statements of transcripts, 80% of agreement between coders is used as a percentage difference target. However, we argue for this result of 78.3% because the 2 coders agreed to share the coding of the 2 full transcripts (about 600 statements), which resulted in minimizing the expected percentage of agreement between the 2 coders.

To ensure that the data collected within qualitative and quantitative research were correctly interpreted by the research team and can be used to build new insights, it is imperative that data analysis be conducted using best practices [29]. These best practices should include methods to safeguard the trustworthiness and quality of the research. According to McAlister et al [30], when one is using qualitative coding techniques, establishing the IRR is a recognized method of ensuring the trustworthiness of the study when multiple researchers are involved with coding. In the course of our study, we used the IRR to test the strength of our categories for our codebook development. Being under the threshold of 80% agreement may indicate that there are categories that need to be further negotiated in subsequent studies. Consequently, in this research, trustworthiness through IRR gauged how well the evidence presented supported the value of the results, while quality analysis measured how likely systematic error and bias were prevented through the design of the study.

### Codebook Development

Our RQ was about HPs’ experience with misinformation and its impact on their professional practice. Two researchers, with shared experiences in collecting and analyzing qualitative data, collaboratively developed the codebook informed by many meetings before, during, and after the data collection to develop the RQs, following up on the flow of the interviews and discussing the emerging themes after the interviews.

A total of 13 interviews were conducted and transcribed. The initial plan was to select randomly 1 (8%) interview of the 13 to share coding and run the IRR exercise. However, running the “Word Frequency” query over the 13 interviews using NVivo (QSR International) identified possible themes, particularly in the early stages of the project [31]. It showed that there were repeated uses of words and their synonyms in the interviews that reflected perspectives of the interviews that underpinned the interviewees’ opinions about misinformation. For example, in the political perspective interview, words such as “political affiliation,” “bias,” “league,” “party,” and “political” were frequently repeated. Therefore, after identifying the 3 main perspectives (professional, social and cultural, and political) both coders agreed to select and code 1 interview from each perspective.

The 13 interviews were then grouped according to these perspectives into 5 professional, 7 social and cultural, and 1 political interview. Next, the researchers selected 1 interview sample that related to each of the 3 themes. In total, 3 interviews were selected to practice the coding together and to conduct the IRR exercise. After that, the 2 coders started to code the 3 selected interviews separately. Following O’Connor et al [32], the researchers identified initial themes through group discussions of overlaps and divergences. Before coding, researchers started to cobuild the baseline of understanding the research aims, RQs, and interviewees’ professional backgrounds, how HPs witnessed misinformation in the job practice, and how to communicate with their patients accordingly. Moreover, in-depth details about the HPs’ job practice and approach to dealing with misinformed patients and the applied approach, training, and resources that the HPs follow or recommend were determined. This phase ended with developing the codebook (see Table 1), followed by the IRR practice (as shown in Figure 2). The structure of the combined deductive and inductive codes is provided in Multimedia Appendix 1. Table 1 shows an example of the developed codebook.

**Table 1:** Example of subthemes in the codebook, definitions, and quote examples.

Code	Definition	Example
Authority	Subcode to the supercode “blind trust” or “status of information and perception	“Sometimes, it could be their religious leaders; sometimes it could be their elders.”
Logic	Statements about patient epistemology relying on logic as “evidence” of misinformation or information	“I think it’s easier when you’re treating somebody from the leafy suburbs of, let’s say, Southampton, where we live, people who are well-off, high up on this economical, know how to search for up-to-date health information, look at the guidelines, and they come armed with a lot of knowledge which then, and you can easily win them over in an argument.”
Availability	Absence of or lack of exposure to verified information leading patients to trust easily accessible information (eg, Google or social media)	“When you don’t have information, you tend to start to pick up sources which are not necessarily valid, not recommended by a health practitioner, or could be related to other parts of the world.” “Sometimes, it’s the patient; they resist the treatment because they, someone from the family, told them that, something.”

## Results

### Data Analysis and Findings

**Figure 3:**
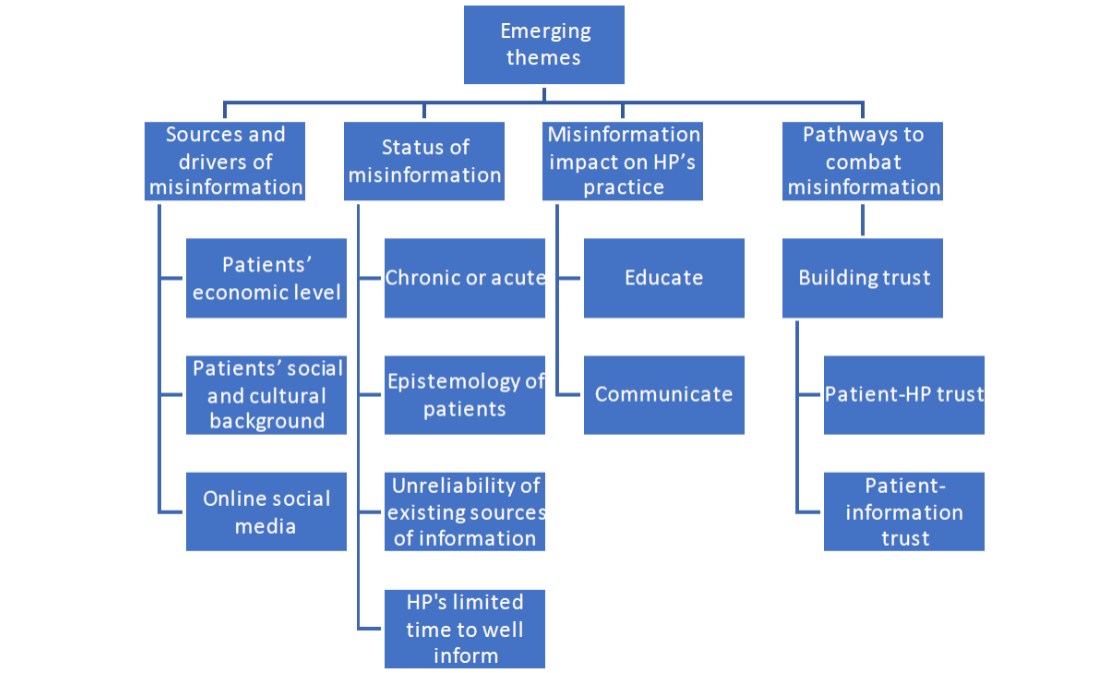
Summary of emerged themes. HP: health professional.

Qualitative data were thematically analyzed to focus on the key aspects related to the study RQs. TA is the search for and extraction of general patterns found in the data through careful reading and re-reading across the qualitative data set [25]. TA flexibility involves making several decisions regarding data collection and analysis before they are undertaken. In this research, the initial analysis started with ongoing discussions between the researchers; these discussions informed the development of the codebook. Within the analysis and flexibility of TA, there were opportunities for newly developed themes (other than those in the codebook) to emerge within the data analysis. TA began while the interviews were ongoing, and transcripts were analyzed one by one using NVivo 12. As the analysis progressed, a figure of emergent codes was developed and refined (see Figure 3). Each new transcript led to codes being further expanded or adjusted. Once all the transcripts were analyzed, researchers refined each code in order to identify any duplicate coding or emerging patterns. Upon completing this process, the themes were developed, refined, and named.

Findings from the analysis of qualitative interviews navigated us on a journey starting from the origins of misinformation (patient’s education, online sources) to the status of misinformation in HPs’ job practice regarding its occurrence—whether it is a novelty or an ongoing phenomenon. The status of misinformation includes how patients think about, accept, and trust this knowledge, highlighting 2 main challenges addressed by HPs that are considered catalysts for the spreading of misinformation. First, existing information is somehow unreliable (eg, out of date). Second, HPs have limited time to meet, listen to, and talk with their patients. For HPs, misinformation impacted their job practice through applying 2 different approaches: (1) educate the patients through multiple methods (eg, HP’s advisory role) and (2) communicate with the patient. However, the pathways to implementing these approaches focus on building patient-HP relationships and building trust between them. “Trust” is an emerging theme that includes patient-HP trust and patient-information trust (see Figure 3).

The following section details the identified themes with supporting quotes.

### Emergent Themes

#### Sources and Drivers of Misinformation

HPs narrated 2 main reasons that are behind misinformation: (1) patients’ backgrounds and (2) the unreliability of the information available. With regard to the patients themselves, reasons such as education, culture, religion, and political affiliation were listed as sources of information.

##### Patients’ Educational Background and Misinformation

Interestingly, according to HP4, the educational level (high or middle level of education) may drive the patients’ information toward 2 alternative routes, either follow or unfollow.

You’ll be surprised to know that…I’m not going to say that the very poor people, but I’m just going to say the working category among the people. They were very keen to follow the right information. And as you go up in the society, when you go to, a little bit higher-educated people; unfortunately, they was the people who was not getting the right information because of their political backgrounds or whatever kind of media they listen to.

HP4

##### Patients’ Social and Cultural Background and Misinformation

Furthermore, the interviews with the HPs revealed the role of the community and word of mouth in spreading misinformation. As emphasized in the following 3 quotes, online social networks create amplification, an illusion of significance, and false credibility for these resources.

We have people from different backgrounds who will look up information in their home countries, which not necessarily could be medical information, but still use them in this country to request for a treatment or trying to sort of push for a particular way of management.

HP9

Well, it is because it has the echo chamber phenomena, which means that you only need 1 or 2 people in the community to relay a message across certain networks, like WhatsApp or whatever. And then suddenly, those messages will amplify themselves like an echo chamber and become so popular and track a lot of...and you see some people resending the messages so many times that the message become significantly more present in those networks from multiple sources, although they might have started with 1 or 2 people. But that then somehow infer [sic] or confirm legitimacy or authenticity on that message and becomes difficult to tackle. Now, because [the] social network has no limits, is like the physical encounter between 2 people, you need to see somebody and let’s say gossip, or misinformation…you have somebody who will pass a message to you, and then you need to wait for another person who to meet to say that message again to you and a third person.

HP5

I think it comes from all sorts of sources. Yeah, so I don’t think it’s always just the internet…think it comes from TV. I think it comes from friends. It comes from family, it comes from generations, it comes from old books. I think it comes from other health care professionals. I think health care professionals within our own spheres are not updated…I think it’s all over the place.

HP12

##### Online Social Media and Misinformation

The social platform, the groups, especially the groups. They are believing them, especially the groups. Some of them, they are dark web, I can see, dark web. And last is Google…because Google gives them the good information.

HP11

We have people who search the internet and come up with all sorts of information, which has not been substantiated in any way and come up requesting a treatment.

HP2

#### Status of Misinformation

##### Misinformation (Chronic or Acute?)

HPs look at misinformation as something more “chronic” rather than “acute.” It reminds us that what makes misinformation during COVID-19 has less to do with how much misinformation is shared and more to do with existing inequalities, cultural exclusion, and lacking communication structures that make the consumers of misinformation more likely to believe and, crucially, act on misinformation. HPs deal with all kinds of health-related misinformation, and it appears to be related to many of the same factors. As stated, “Misinformation is always there, not only because of the pandemic” (HP10).

##### Unreliable Sources of Misinformation

Asked whether patients would have access to the right information, HPs provided varying responses regarding information resources. Although some health care professionals described the resources of information as being available, they also highlighted the lack of trust patients have in them. This contrasts with the high level of trust placed in social media. However, some HPs have frequently complained that the misinformation is based on outdated sources.

Some of them [is] outdated information, where people have been not aware of new developments on treatments. So that’s…we can call it misinformation or lack of information. And then you have people who come up with some ideas about different kinds of treatments or lack of treatment.

HP2

It is important to mention that a few HPs addressed the difficulty of facilitating this reliable information.

I think it’s very difficult to give people key health messages that are current, up to date, and researched and consistent.

HP3

Yes. So, when you don’t have information, you tend to start to pick up sources which are not necessarily valid, not recommended by a health practitioner, or could be related to other parts of the world.

HP9

##### Epistemology of Patients

According to many HPs, patients blindly trust some of the sources of misinformation. This blind trust is based on 3 factors: authority of the sources, availability, and logical reasoning (plausibility).

###### Authority

Using the following example, HP2 explains that when the information is provided by a “gatekeeper” (a native speaker of the patient’s own language), it is considered more authoritative than information provided by an “outsider” (a nonnative speaker).

So, the guy was translating my chat in Turkish because he knew English and he was very respectful and very appreciative. And he said, “My apologies for having to second, remention your message.” And he knew that a lot of them knew English. And they could know what I’m saying. But they wanted to hear it in Turkish from somebody who’s in Istanbul, while they are in England.

HP2

###### Availability

The availability of information (potentially in the absence of, or lack of exposure to, verified information) is 1 of the reasons why patients may choose to trust information to which they have easy access. There are many people who share information online (eg, from Google or social media), and the accessibility of this information is attractive, especially when the HP is difficult to reach.

I think they just follow whatever suggestion is given by the search engine, and later on, just after the consultation and coming across to the specialist, they start to be aware of the actual accredited body of the society of the disease that they are dealing with.

HP3

###### Logic

It is clear from the narrative that patients blindly trust the information when it occurs logically in the context of the community surrounding them and when it aligns with their experiences and perspectives. The patient, for example, anticipates that, since their friend had an illness, they will also have a similar experience.

She has explained to me that she has had these symptoms for a while, and I was like, “So what makes you think now this is the problem?” and she was like, “Well, my friend just recently got diagnosed and she has had this for a while as well,” and she said she’s got some of the symptoms her friend has, so it’s not even like she completely has what her friend has…she’s like, “Oh, yeah, it’s just the painful periods and the heavy periods.”

HP7

Again, same problem, irregular periods, but this time her friend had just been diagnosed…or they had found cysts on her ovaries, so she has been diagnosed with polycystic ovaries, and she was like, “Yeah, she’s the same age as me. I have really painful periods as well and this must be it,” even though this has been the situation, it’s not like it has gotten worse over time, it’s just…yeah, they will have a conversation with someone, and they are like, “Oh, this is what’s happening, this makes sense,” and it is kind of confirmed when they search Google, as well.

HP2

#### HP’s Limited Time to Well Inform

You [HP] will take a lot of time, time they visit or make an appointment, meet their GP if they do not have this time they may become more inclined to rely on other sources of information.

HP10

### Misinformation Impact on HPs’ Practice

#### Educate the Patient

HPs adopted 2 approaches to dealing with misinformation. A majority of HPs agreed on the first point, which is to educate the patients. HPs use different strategies to educate patients, such as instructing, patronizing, presenting evidence, coaching, or researching the patient to determine the appropriate channel to use. Nevertheless, it has been observed that some doctors patronize their patients and treat them with an air of superiority. As part of a treatment path, other strategies may also be used, such as setting aside time to listen, talk, and communicate and building trust.

“You go online and look it up yourself,” which obviously sometimes, because I wouldn’t give them a specific website. I’d just say, “Look it up yourself for head injury advice.”

HP8

Actually, just you explain that you understand the culture, but this is totally different of this, and this is classic mental illness. And we just do like psychoeducation about the signs and symptoms of mental illness and how we treat this. And we try to convince them and, like, reassure them about the medication. It’s not causing addiction. It’s not severely affecting the patient. Just, we are trying to treat them and make them feel better and make them more functioning.

HP10

If you are in a very busy inpatient ward, it’s a very heavy workload, but still if you have 1 patient who is refusing treatment, it is worth to go and talk to him face to face. Sometimes, the patients refuse to do, like, a blood test, or sometimes, they are refusing to go to the general hospital if they are having high blood sugar with ketones and they are going to diabetic ketoacidosis, so if I talk to him, I will save his life, literally. So, it’s worth doing it even if you have something else.

HP10

I would probably take more time with them, because I think it would take more…you maybe would have to spend more time. And sometimes, there would be resistance, I think, to maybe, you’re telling them something that is the opposite to what they’ve been told, possibly by somebody that they would trust, or somebody...We do get doctors telling people the opposite of what we say sometimes. And then having to kind of change that advice is quite difficult. So, a doctor may say to somebody, “You must change your catheter leg back(?) every day or something, for example. And actually, you only change them once a week. And so, if a doctor has told somebody something, misinformed them, and then a nurse who is seen as a, you know, of a lesser.

HP12

But I think sometimes, certain treatment, from my experience from going on insulin, you can tell that people from, for example, [an] Asian background, they just have lots of concern about going on insulin, because they have heard so many stories, or they think that going on insulin doesn’t mean they are really badly with diabetes, they don’t want to accept that. So, this is 1 example, for example, changing the treatment from tablet to insulin, you just see lots of resistance from some patients. For example, like 1 patient told me, “Oh, my cousin lost her leg after she went on insulin.” But actually, just when I explained, “No, it’s not when she went on insulin. It’s because the diabetes wasn’t treated well.”

HP1

#### Communicate With the Patient

HPs highlighted the importance of communication with the patient as an important pathway in their job practice to spot and witness misinformation, know its drivers and sources, and find pathways to confront it.

There’s something called Sugar Buddies that if somebody was type 1 diabetes can be paired with somebody who had type 1 diabetes for 20 years, and really very well insulin management, so we pair them with somebody who we know that he’s been really very well educated, managing. So, that has been helpful as well. And then the patient[s] like it, and they actually meet other patient[s] with the same condition.

HP2

For interviewees, communication has been addressed differently. For some, it is to talk and provide information; for others, it is to listen to the patient and discuss further health-related topics that have been spotted misinformed. As stated by HP3 and HP8:

It is how you communicate with people who are coming up with these kinds of challenges.

HP3

It is down to communication skills. Adapting maybe my tone, my voice, my speech, my physical gestures to a patient.

HP8

Interestingly, the words “listen” and “listening” to patients as part of the role of HPs were mentioned in the narrative 60 times across the 13 interviews. For the majority of interviewees, time is a common requirement to communicate with their patients.

##### Time Is a Requirement to Communicate With Patients

I would have to spend some more time building some trust and showing that you know what you talk about. Because I think sometimes, people, when they see that you know what you’re talking about, they will see that actually after some time, then they will build some trust in you. But sometimes, yeah, so you probably would spend more time with that person. And feel you needed to build some trust and get them to, you know, invest in your point of thinking.

HP10

You will take a lot of time. And not only 1 session…also you should…this is really my practice is like that, it is not from the first take, you will never convince him from the first take, you should give him the basic information first, let him think about, then another talk with him at another, you know, in another situation, and in another meeting. They are partly convinced when you speak, you know, more frequently about it—in many sessions, not only 1 session. And don’t give the final decision from 1 session or 2 sessions; you should give him more time to digest this information.

HP11

### Pathways to Combat Misinformation

#### HP-Patient Relationship

Misinformation is often influenced by the HP-patient relationship, whether it is short term or long term. For instance, if patients speak to a different health care provider each time they visit or make an appointment, if they do not have a family doctor, they may become more inclined to rely on other sources of information.

I think so, I think so, just because when you are in the [general practice], it’s quite early on, and the GP [general practitioner] is kind of a filter as well, that’s how you know who needs to be passed on to secondary care, and who has just got very severe health anxiety, and they need someone to sit and talk to them. You can even harshly sometimes just tell them, “You’re okay, go away.” Even with the GPs, sometimes, they have to do that, over the phone. So, they’ve had like diarrhea for a day, or they had diarrhea this morning, it’s like, “Why are you calling, we all get diarrhea” kind of thing…do you know what I mean? They’re like…I think they even quote things from Google, like, “This is malabsorption.” The GP is, “Do you know what malabsorption is?” Do you know what I mean? So, I think GP, you get a lot of that stuff, but you kind of have to sieve through it, and sometimes you have to be like, “Stop, you’re overreacting” kind of thing.

HP3

Patients need to see a face; they need to see somebody on their territory or in areas like social networks they have. If they have a Teams or a Zoom or a Skype meeting or a function or whatever, you need to show some presence there so that you then...because as a clinician, we don’t treat people virtually. We have to, at some point, meet them and give them the treatment. So, you need to be a physical presence in their life at some point.

HP2

There is a long-term investment; [a] short-term one will be to make sure that your message is clear and short and concise. And the medium term will be to get access to networks which those communities are using through your liaisons…are your champions in that community who can then give you some access into them so that you can pass on the messages into those communities, either directly or through proxies, through them to counteract any kind of misinformation.

HP1

It is worth to go and talk to him face to face. Sometimes the patients refuse to do, like, a blood test, or sometimes, they are refusing to go to the general hospital if they are having high blood sugar with ketones and they are going to diabetic ketoacidosis, so if I talk to him, I will save his life, literally. So, it’s worth doing it even if you have something else.

HP10

#### Building Trust

Trust was 1 of the strong themes in the study findings; it was cross-referenced across several interviews. It included different parties—the trust between the HP and the patient and the trust of the health system itself, including the existing information (eg, websites and leaflets).

##### HP-Patient Trust

If the patient can trust me, he would be more convinced when I talk to him about the misinformation that he had and about the right information if we can see(?) or the valid information about his disease and the treatment options. So, if I manage to get him trusting me, it would make a big step in our relationship. But the problem is a lot of our patients have no insight at that time. A lot of our patients are being paranoid.

HP10

##### Trusting the Health System Information

For combating the misinformation, but increasing the credibility and the trust of your local health system here, more representation from those communities within the health system, within the hierarchy as well. So, it’s not only a global level, even at managerial, and where they can see that our people are in high positions, and they are endorsing a message of health or health awareness or a treatment or a campaign on increasing information on this and that. So, this will then allow them to drop those misinformation.

HP2

## Discussion

### Principal Findings

#### How Do Health Professionals Respond When Experiencing That a Patient Has Been Misinformed?

According to the study findings, during the conversation between HPs and their patients, HPs explore the sources of information. For them, identifying the origin of this information helps them identify the impact and consequences of the information and find pathways to confront the misinformation. For example, if the patient is a social media follower, that informs the HP to provide online accredited sources instead of the misinformative followed source [33]. Therefore, identifying sources and drivers of misinformation is considered a start to the pathway to confront misinformation [33].

The second manifested response to misinformation is how HPs see the misinformation: as chronic and as ongoing.

#### What Misinformation Has the Greatest Impact on Medical Practice?

For the second RQ in this study, which questions what HPs feel the biggest impact of misinformation is on health care practice.

The study does not specify certain misinformation that is considered to be leading to the biggest impact. HPs deal with all kinds of health-related misinformation, and it appears to be related to many of the same factors. For HPs, it all affects health and it is all big. This may be because according to the collected data, patients are misinformed because of the implications of different and diverse sources, such as online (eg, Google), family members, and offline (eg, social communities). It could also be that misinformation is an ongoing issue and is not linked with the pandemic solely. For HPs, all types of impacts of misinformation are major and need almost the same pathways to combat. For example, misinformation negatively impacts chronic patients (eg, those with bowel cancer). Misinformed patients resist following up on the chemotherapy protocol recommended by the HP.

#### What Aspects of Change and Intervention in Health Professionals’ Practice Are in Response to Misinformation?

##### Educate the Patient

Lilley [33] confirmed that there is a need to educate patients to prevent errors and improve the quality of health care. This education influences patient behavior and produces the changes in knowledge, attitudes, and skills necessary to maintain and improve health. However, educating patients is not an easy and direct job. According to Ward et al [34], it is a myriad of interventions to support patients’ education and adherence to doctor or HP recommendations for diet, exercise, medications, and advice. This conclusion is well aligned with what HPs narrated in this study, as they use different methods to educate their learners, including being advisors, providing resources, and acting as tutors and counsellors.

##### Communicate With the Patient

According to Palmieri and Stern [35], effective bidirectional communications (between the patient and the HP) are pathways to making an accurate diagnosis. The impact of communicating with the patient is not limited to the issue of

misinformation, but it would lead to better health service provision to the patient. According to Davis [36], a patient who listens and has ongoing communication with the HP (eg, doctor) first and then decides has done a better job of deliberating than a patient who first consults web pages or friends and acquaintances and makes a decision before talking to the doctor. Such patients are simply not as good at deliberating about medical matters as patients who do not engage in premature consent. Knowing which sources to heed when making medical decisions is part of being competent at making such decisions. It is part of the skill of deliberation. The challenge to address in relation to encouraging communication is the limited time that HPs can allocate to meet, talk, and listen to their patients. This is confirmed by Palmieri and Stern [35] as managing care and time constraints, adding further pressure on HPs that need to be facilitated. Communication is a 2-way effort to maintain, and there is a role for each party [37]. HPs are encouraged to rehearse different communication strategies and to seek supervision and consultation around matters that are challenging. Patients have a role in fostering honest communication with their providers, while physicians can best promote such interactions by being thoughtful, deliberate, and self-aware.

#### What Is the Intervention in Health Professionals’ Practice in Response to Misinformation?

For this third RQ, according to the study findings, trust is the dimension that needs to be tackled to combat misinformation. In the issue of trust, the study encompasses 2 aspects: patient-HP trust and patient-source of information trust that is issued by a health body, such as the World Health Organization (WHO).

##### Building Trust

Trust involves relationships and not just facts. Trust is most likely in situations in which people directly encounter a health care professional in person (at least virtually) rather than in situations in which people are presented with information in other ways [38]. HPs who spoke about gatekeepers from the different communities they serve, or building long-term relationships with patients, understand that many patients will trust people they know—people who share the same language or cultural experiences. Making inroads with different communities and representing them in health care are strategies that can mitigate the impacts of misinformation. This is a time-consuming activity, but it has a long view, and this is important. If HPs view misinformation as chronic, then they understand that the treatment pathway must be more in depth and contextualized.

##### Patient-HP Trust

Regarding the personal characteristics or demographic features that may play a role, HPs named education, culture, and political affiliation as playing a role. This is consistent with the existing literature. However, similar to Harper and Baguley [20], the correlations are not always in 1 specific direction (more or less related to misinformation-sharing behavior). What appears to be important is how they impact trust. If patients have a trust relationship with their health care provider, and trust in their relevant authorities to provide accurate and timely information, it may not matter how much misinformation a person sees. They will be able to make good decisions for their health and take the necessary actions to protect themselves and their families.

##### Patient-Source of Information Trust

HPs in the study findings linked misinformation to trust, both between patients and their health care providers as well as between patients and the information provided by experts (eg, health organizations and accredited websites). Some of these official resources do not provide up-to-date information. Although this issue does not directly relate to HPs, it affects their practice in terms of patient-information trust. This mistrust is confirmed by Davis [36], as the rise of premature consent cases is that trust in the health care system has been undermined by instantaneously disseminated information about medically related errors without any details. Making an accurate diagnosis relies on the provision of reliable information [35]. Nonetheless, this information leads the tech-savvy patient to be skeptical about the physician-employee’s management. Therefore, evaluating sources of medical information and advice are pathways to support patients to decide whether to believe and trust the provider.

### Limitations

The study had 2 main limitations: difficulty in recruiting participants and the lack of patients’ voices.

#### Recruitment of Participants

This study was conducted during the pandemic when the majority of HPs were extremely busy and overloaded with work duties and pressure. Consequently, recruitment of a satisfactory number of participants to take part was 1 of the main limitations of this study. The further difficulty, after finding HPs willing to be interviewed, was to find a time slot in their agenda to schedule the interview in the middle of a global pandemic.

#### Patient’s Voice

Although HPs in this study could give an in-depth view about their patients’ misinformation, this is considered to be only 1 side of the coin. The other important aspect that needs to be explored in depth are the patients’ views about misinformation, including its drivers, perception, and how to confront it from their angle. With the limited time and funding for this pilot, we thought it would be more effective to speak to HPs about many different diverse experiences with patients, rather than collecting a small sample of patient views, at this time.

### Future Work

Many HPs recommended that this study be complemented with another empirical study that incorporates patients’ voices and explores their views. According to several interviewees, patients’ views, understandings, beliefs, and attitudes can shed light on different angles from those narrated by HPs. Including those views will aid in understanding misinformation more thoroughly and in depth. However, recruitment of patients who have experienced misinformation might be a challenge in this study, because it may be difficult for some individuals to admit that they have been misinformed.

### Conclusion

Misinformation affects patients’ decisions to follow a treatment or guidance prescribed by their HPs. According to the study findings, patients follow misinformation resources for 3 reasons: (1) available resources (eg, Google); (2) meaningful resources, as they reflect their personal or cultural beliefs; and (3) authorized resources, as they have been disseminated by a source of power for the patient (eg, a political party). The qualitative research presented in this paper revealed that patients do not always trust their HPs or the authorities about health-related information. As a result, they may choose not to follow HP advice on matters that impact their health, including COVID-19. The lack of trust in HPs was identified as a prominent theme in this study, and it was attributed to several factors, including trusting other sources of information (eg, social media), patient’s doubts about HP experience (eg, a junior doctor with only a few years’ experience), and patients’ doubts about the available sources of information that are provided by the HP (eg, out-of-date resources). There are 2 dimensions of trust: patient-HP trust and patient-information trust. There are 2 necessary actions to address the issue of lack of trust in these dimensions: (1) building trust and (2) maintaining trust. The main recommendations of the HPs are to listen to the patients, to give them more time, and to seek evidence-based resources. Finally, misinformation is an ongoing phenomenon; it is not solely manifested during the pandemic and the spread of fake news where some patients resisted COVID-19 vaccination. Misinformation has been shown for patients with other chronic diseases (eg, bowel cancer). These patients, because of misinformation, resisted following up the chemotherapy protocol recommended by the HPs. Consequently, for HPs, finding out the sources and drivers of misinformation is a pathway to identify, track, and confront misinformation.

## Appendix

Multimedia Appendix 1 Structure of combined deductive and inductive codes.

## Abbreviations

HP: health professional
IRR: interrater reliability
NHS: National Health Service
RQ: research question
TA: thematic analysis
