# Supplementary material for: The Experience of Health Professionals With Misinformation and Its Impact on Their Job Practice: Qualitative Interview Study
Source: JMIR Form Res. 2022 Nov 2;6(11):e38794. doi: 10.2196/38794 (PMC9635441; doi:10.2196/38794)
Supplement: Multimedia Appendix 1 [file formative_v6i11e38794_app1.docx]

Structure of combined deductive and inductive codes

- **Status of information**
  - Out-of-date
  - Unavailable
  - Unfollowed
  - Untrusted
  - Maintenance (e.g., repetition)
  - Blind trust
  - Authority
  - Logic
  - Availability
- **HPs’ estimation of misinformation**
  - Misinformation is chronic
  - Misinformation is acute
- **Influential factors for treatment pathway**
  - Diversity of patients
  - Patient-doctor communication
  - Short-term relation
  - Long term relation
  - Space and Physicality
  - Values & beliefs (politics, social, cultural)
- **Treatment**
- **Patient education**
  - Instructive
  - Evidence-based
  - Guidance/coaching
  - Patronising
  - Avoid patronising
  - Advisory
  - Research the patient
  - Treatment-resources
  - Time
  - Communication
  - Listen
  - Trust
  - Building trust
  - Gate openers
  - Marinating trust
- **Challenges**
  - Limited Time
  - Workload
  - HP disagreement of views
  - Social media (echoing, confusing, etc.)
